# Eugenol Induces Phenotypic Alterations and Increases the Oxidative Burst in *Cryptococcus*

**DOI:** 10.3389/fmicb.2017.02419

**Published:** 2017-12-07

**Authors:** Júnia C. O. Alves, Gabriella F. Ferreira, Julliana R. Santos, Luís C. N. Silva, João F. S. Rodrigues, Wallace R. N. Neto, Emmanueli I. Farah, Áquila R. C. Santos, Brenda S. Mendes, Lourimar V. N. F. Sousa, Andrea S. Monteiro, Vera L. dos Santos, Daniel A. Santos, Andrea C. Perez, Thiago R. L. Romero, Ângelo M. L. Denadai, Luciana S. Guzzo

**Affiliations:** ^1^Faculdade de Ciências da Saúde, Universidade Vale do Rio Doce, Governador Valadares, Brazil; ^2^Departamento de Farmácia, Universidade Federal de Juiz de Fora – Campus Governador Valadares, Governador Valadares, Brazil; ^3^Centro de Ciências da Saúde, Universidade CEUMA, São Luís, Brazil; ^4^Instituto de Ciências Biológicas, Universidade Federal de Minas Gerais, Belo Horizonte, Brazil

**Keywords:** eugenol, *Cryptococcus gattii*, *Cryptococcus neoformans*, antifungal activity, cutaneous cryptococcosis

## Abstract

Eugenol is a phenolic compound and the main constituent of the essential oil of clove India. Although there are reports of some pharmacological effects of eugenol, this study is the first that proposes to evaluate the antifungal effects of this phenol against both *Cryptococcus gattii* and *C. neoformans* cells. The effect of eugenol against yeast cells was analyzed for drug susceptibility, alterations in cell diameter, capsule properties, amounts of ergosterol, oxidative burst, and thermodynamics data. Data demonstrated that there is no interaction between eugenol and fluconazole and amphotericin B. Eugenol reduced the cell diameter and the capsule size, increased cell surface/volume, changed positively the cell surface charge of cryptococcal cells. We also verified increased levels of reactive oxygen species without activation of antioxidant enzymes, leading to increased lipid peroxidation, mitochondrial membrane depolarization and reduction of lysosomal integrity in cryptococcal cells. Additionally, the results showed that there is no significant molecular interaction between eugenol and *C. neoformans*. Morphological alterations, changes of cellular superficial charges and oxidative stress play an important role in antifungal activity of eugenol against *C. gattii* and *C. neoformans* that could be used as an auxiliary treatment to cutaneous cryptococcosis.

## Introduction

Cryptococcosis is a systemic mycosis caused mainly by *Cryptococcus neoformans* and *C. gattii* species (Hagen et al., 2015). Disseminated cryptococcosis is the worst prognostic of the disease, and can affect several organs beyond lungs, such as the central nervous system and skin (Chayakulkeeree and Perfect, 2006). Pathological manifestations in the skin, that occurs in 10–20% of patients, are characterized by different clinical features including ulcers, subcutaneous nodules, and cellulitis (Chayakulkeeree and Perfect, 2006; Capoor et al., 2008; Ikeda et al., 2014). The current antifungal therapies used for cryptococcosis such as amphotericin B and azoles have certain limitations due to side effects and emergence of resistant strains (Gast et al., 2013; Scorzoni et al., 2017). An alternative strategy for the treatment of cutaneous and subcutaneous cryptococcosis would be the application of formulations based on natural products derived from plants such as essential oils (Lemos et al., 2005; Baptista et al., 2015). In general, essential oils are complex mixtures of organic compounds, like diterpenes, monoterpenes, sesquiterpenes, and phenylpropanoids (Tadić, 2012; Gazim et al., 2014).

Phenylpropanoids are antimicrobial compounds characterized by a phenyl ring bearing a propenyl side chain (Louie et al., 2007). The eugenol [4-allyl-2-methoxyphenol] is the phenylpropanoid most widely studied for various biological activities (Louie et al., 2007; Carrasco et al., 2012; Perugini Biasi-Garbin et al., 2015). Previous research has shown that eugenol is a potent antifungal and antibacterial agent, and its antimicrobial action seems to be related to the inhibition of electron transport and perturbation of permeases in the cytoplasmic membrane (Gill and Holley, 2004; Darvishi et al., 2013). However, studies have shown that eugenol is related to the generation of oxidative stress concomitantly with lipid peroxidation of the cell membrane of *Candida albicans* yeast, and the generation of reactive oxygen species (ROS), that can also contribute to cell death (Khan et al., 2011).

Thus, based on the fact that eugenol is already well-described in literature as a potent antifungal and antibacterial agent, the aim of the present work was to investigate the antifungal effect of this phenol against both *C. gattii* and *C. neoformans* cells.

## Materials and Methods

### *Cryptococcus* Strains and Study Design

For assays “Antifungal drug susceptibility testing” and “Time-kill curves”, we tested two reference strains of *Cryptococcus gattii* (ATCC 24065, ATCC 32608) and three reference strains of *Cryptococcus neoformans* (ATCC 24067, ATCC 28957, ATCC 62066) that were obtained from the Culture Collection of the University of Georgia (Atlanta, GA, United States). Six clinical isolates of *C. gattii*, five clinical isolates of C. *neoformans*, and one environmental isolate of each species, all from the Culture Collection of the Mycology Laboratory/ICB-UFMG, were also used in this study (Magalhães et al., 2013). Isolates were maintained on Sabouraud dextrose agar (SDA) at 4°C. Prior to each test, the strains were subcultured on SDA for 48 h at 37°C. Two reference strains of each species (*C. gattii*: ATCC 24065, ATCC 32068; *C. neoformans*: ATCC 28957, ATCC 62066) were chosen for further experiments.

### Antifungal Drug Susceptibility Testing

The minimum inhibitory concentration (MIC) for eugenol (Sigma–Aldrich, St. Louis, MO, United States) was determined by the antifungal microdilution susceptibility standard test proposed by the CLSI M27-A3 method (Institute Clinical and Laboratory Standards, 2008). The inoculum was prepared in sterile saline and the transmittance of the suspensions was adjusted to 75–77% (530 nm), followed by further dilution in RPMI-1640 buffered with MOPS (Sigma–Aldrich, St Louis, MO, United States) medium to achieve 1.0 × 10^3^ to 5.0 × 10^3^ CFU/mL. The final concentrations ranged from 2 to 1024 mg/L for eugenol. The plates were incubated at 35°C for 72 h. The MIC for eugenol was determined visually as 100% growth inhibition when compared to the control. The results were confirmed by adding the salt 3-(4,5-dimethylthiazol-2-yl)-2,5-diphenyl-2H-tetrazolium bromide (MTT) (Sigma–Aldrich, St Louis, MO, United States) (5.0 mg/mL) to determine the reduction in the metabolic cell activity. Briefly, the plates were incubated at 35°C for 3 h and DMSO was added before spectrophotometric reading at 490 nm. The MIC endpoint for interpreting the results was 100% of reduction in metabolic activity for eugenol compared with the control. The isolate *Candida parapsilosis* ATCC 22019 was used as a quality control. All the tests were performed in duplicate for each strain.

### Time-Kill Curves

An assay was performed to evaluate the time-kill kinetics of the drugs against *C. gattii and C. neoformans* strains, as described previously (Ferreira et al., 2013). For eugenol, the concentration tested was equivalent to the MIC (256 mg/L – *C. gattii*: ATCC 24065, *C. neoformans*: ATCC 28957, ATCC 62066; 64 mg/L – *C. gattii* ATCC 32068) or twice the MIC (2× MIC). Hundred microliter aliquot was taken from the microtitre plates containing the yeasts against the MIC and 2× MIC of eugenol at different intervals until 72 h and metabolic activity had been measured using MTT, as described above. The percentage of metabolic activity compared with control growth was determined for each strain for each time of reading. The results were confirmed by plating a quantity of each well on SDA and incubating at 37°C for 72 h for colonies counting.

### *In Vitro* Interaction of Eugenol with Fluconazole and Amphotericin B

Eugenol was tested in combination with fluconazole and amphotericin B, the drugs usually chosen in cryptococcosis treatment. A checkerboard microdilution method, which provides a matrix of all possible drug combinations in the required concentration range, was used to test the susceptibility of the four *Cryptococcus* strains (two *C. gattii* and two *C. neoformans*) to the drugs. The eugenol concentrations and inoculum were prepared as described above. The concentrations of fluconazole and amphotericin B ranged from 0.125 to 64 mg/L. A volume of 100 μL of the inoculum suspension was transferred to sterile flat-bottom 96-well plates containing 50 μL of each of the antifungal drugs and 50 μL of eugenol or RPMI-1640 (control growth). The plates were incubated at 35°C for 72 h. The cellular metabolic activity was determined using MTT salt. MIC endpoint analysis of the results was 50% metabolic activity reduction for fluconazole and 100% metabolic activity reduction for amphotericin B compared to the drug-free control.

The interaction between drugs was quantitatively evaluated by determining the fractional inhibitory concentration index (FICI) (Odds, 2003)^.^ The formula for calculating FICI was: FICI = [MIC Fluconazole or Amphotericin B in combination with eugenol /MIC Fluconazole or Amphotericin B alone] + [MIC eugenol in combination with Fluconazole or Amphotericin B/MIC eugenol alone]. FICI was calculated for all the possible combinations of different concentrations. The interaction between these drugs was classified as synergism if FICI ≤ 0.5, indifferent if 0.5 > FICI ≤ 4.0, and antagonism for FICI > 4.0. This assay was tested in duplicate and it was repeated twice.

### Sorbitol Test

Sorbitol is used in a protection assay to provide clues if a drug interferes with the integrity of the cell wall, since it is an osmotic protector (Carrasco et al., 2012). A checkerboard microdilution method had been used to test the interference of sorbitol in the susceptibility of the four *Cryptococcus* strains (two *C. gattii* and two *C. neoformans*) to the eugenol. In this test, it was determined FICI eugenol with sorbitol as described above. The concentrations of sorbitol ranged from 0.05 to 0.8 mol/L and of eugenol ranged from 16 to 1024 mg/L. The reading was performed visually. All tests were performed in duplicates for each strain.

### Ergosterol Quantification

*C. gattii* and *C. neoformans* strains were cultured on SDA (37°C for 72 h) and treated with eugenol at MIC concentrations (MIC eugenol = 256 mg/L for *C. gattii* ATCC 24065, *C. neoformans* ATCC 28957 and *C. neoformans* ATCC 62066; 64 mg/L – *C. gattii* ATCC 32068) and fluconazole (positive control) for 1 h at 37°C. A growth control was also performed. After incubation, the tubes were centrifuged (Jouan, model BR4i) at 1,643 *g* for 5 min at 4°C and the supernatant was removed. The cells were washed with sterile distilled water and the net wet weight pellet was determined, as described previously (Ferreira et al., 2013).

For the extraction of lipids, 3 mL of an ethanolic solution of potassium hydroxide 25% was added to each cell mass and agitated for 1 min. The tubes were incubated in a water bath at 85°C for 1 h and further cooled at room temperature. A mixture of 1 mL of sterile water and 3 mL of *n*-heptane (Sigma–Aldrich, St Louis, MO, United States) was added, followed by agitation in a vortex for 3 min. The supernatant was removed, and the reading was performed in a spectrophotometer at 282 and 230 nm. A calibration curve with standard ergosterol (Sigma–Aldrich, St Louis, MO, United States) was constructed and used to calculate the quantity of ergosterol (Olsson et al., 1987; Santos et al., 2012). In all cases (ergosterol content from yeasts and standard ergosterol), the absorbance of ergosterol was the result from the subtraction of the absorbance obtained at 282 nm and absorbance obtained at 230 nm. Ergosterol and 24(28) dehydroergosterol (DHE), a late sterol pathway intermediate, absorbed at 282 nm, but at 232 nm the amount of absorption due to 24(28) DHE only (Breivik and Owades, 1957). The results were expressed as percentage of ergosterol in comparison with the growth control and represent the means of three independent experiments.

### Measurement of Mitochondrial Membrane Potential and Lysosomal Membrane Stability by Flow Cytometry

Acridine orange (AO) (Olsson et al., 1987) and rhodamine 123 (Rho 123) (Ronot et al., 1986) were used as fluorescent probes for determination of lysosomal membrane stability and mitochondrial membrane potential (ΔΨm), respectively. For both assays, cells from each strain were resuspended at a density of 1 × 10^6^ cells/ml in 500 μL in RPMI-1640 medium supplemented with MOPS. Eugenol was added at the concentrations of 1× MIC and 2× MIC, and the cells were incubated for 1 h (MIC) and 12 h (MIC and 2× MIC) at 37°C (MIC eugenol = 256 mg/L for *C. gattii* ATCC 24065, *C. neoformans* ATCC 28957, and *C. neoformans* ATCC 62066; 64 mg/L – *C. gattii* ATCC 32068). After incubation, the cells were washed three times with PBS buffer (pH 7.2) under centrifugation at 6,000 rpm for 10 min. After washing, the cells pellets were resuspended in PBS (500 μL) and labeled with AO (1 μg/mL in the dark for 20 min) or Rho 123 (10 μg/mL in the dark for 10 min). After incubation, cells were washed three times with PBS. The cells were resuspended in PBS and analyzed by flow cytometry (BD Accuri^TM^, United States; FL3 channel for AO and FL1 for Rho123). A total of 10,000 events were analyzed for each sample. Changes in the fluorescent intensity of Rho 123 were quantified using the variation index (VI) obtained by the equation (MT-MC)/MC, where MC is the mean of fluorescent intensity of control and MT the mean of treated cells. Negative values of VI correspond to mitochondrial membrane depolarization.

### Fluorescent Microscopy

Cells from each strain were resuspended at a density of 1 × 10^6^ cells/mL and treated with eugenol was added at the concentrations of 1× MIC for 1 h at 37°C. After incubation, the cells labeled with AO (1 μg/mL in the dark for 20 min) and recorded images (40×) by fluorescence microscopic system (Axio Imager Z2, Carl Zeiss, Germany) using 495–555 nm band pass (green) filter. Loss of lysosomal integrity can be observed as a rise in green fluorescence.

### Lipid Peroxidation Assay

*C. gattii* and *C. neoformans* strains were cultured on SDA (37°C for 72 h) and treated with eugenol at MIC concentrations (MIC eugenol = 256 mg/L for *C. gattii* ATCC 24065, *C. neoformans* ATCC 28957 and *C. neoformans* ATCC 62066; 64 mg/L – *C. gattii* ATCC 32068) and hydrogen peroxide (positive control) for 1 h at 37°C. The products of the lipid peroxidation were measured as thiobarbituric acid-reactive substances (TBARS) (Ferreira et al., 2013). The pellet was frozen and homogenized with 1 mL in ice cold 1.1% phosphoric acid. Four hundred microliter of the homogenate was mixed with 400 μL of 1% thiobarbituric acid (Sigma–Aldrich, St Louis, MO, United States) prepared in 50 mM NaOH containing 0.1 mM butylated hydroxytoluene and 200 μL of 7% phosphoric acid (all the solutions were kept on ice during manipulation). Subsequently, samples (pH 1.5) were heated for 60 min at 98°C and 1500 μL of butanol was added. The mixture was mixed vigorously using a vortex and centrifuged for 5 min at 2000 *g*. The organic layer was transferred and the absorbance at 532 nm was measured (Termo Scientific Multiscan spectrum, Termo Ficher Scientific). The thiobarbituric acid solution was replaced by 3 mM HCl in the blank controls. TBARS values were calculated using the extinction coefficient of 156 mM^-1^cm^-1^ and represent the mean of three independent experiments.

### Measurement of ROS Production

Endogenous ROS measured by fluorometric assay with specific probes (Ferreira et al., 2013). Yeast cells from *C. gattii* and *C. neoformans* cultured on SDA (37°C for 72 h) were treated with (MIC eugenol = 256 mg/L for *C. gattii* ATCC 24065, *C. neoformans* ATCC 28957, and *C. neoformans* ATCC 62066; 64 mg/L – *C. gattii* ATCC 32068) and hydrogen peroxide (positive control) for 1 h in RPMI without phenol red (Sigma–Aldrich, St Louis, MO, United States) and incubated with 10 μM of 2′,7′-dichlorofluorescin diacetate (DCFH-DA; Invitrogen, Life Technologies, Carlsbad, CA, United States) for ROS quantification. A growth control was also performed. The fluorescence was measured with a Fluorometer (Synergy 2 SL Luminescence Microplate Reader; Biotek) using excitation and emission wavelengths of 500 nm. The results were expressed as arbitrary units of fluorescence ±SEM. These tests were performed in triplicate.

### Antioxidant Enzymes Peroxidase and Superoxide Dismutase Activities

Prior to the tests, a cell-free extract from yeast cells cultured on SDA (37°C for 72 h) and treated with eugenol (MIC eugenol = 256 mg/L for *C. gattii* ATCC 24065, *C. neoformans* ATCC 28957, and *C. neoformans* ATCC 62066; 64 mg/L – *C. gattii* ATCC 32068) and hydrogen peroxide (positive control) was prepared and used for assessing peroxidase (PER) and superoxide dismutase (SOD) activities according to the method described previously (Ferreira et al., 2015). Untreated cells were used as control. Soluble protein was determined using the Bradford test using a standard curve of bovine serum albumin.

### Cell Diameter, Capsule Size, and Zeta Potential Measurements

Yeasts cells cultured with 0.5xMIC (MIC eugenol = 128 mg/L for *C. gattii* ATCC 24065, *C. neoformans* ATCC 28957, and *C. neoformans* ATCC 62066; 32 mg/L – *C. gattii* ATCC 32068) of eugenol were visualized with an optical microscope 100× (Axioplan; Carl Zeiss, Germany) following suspension in India ink. Capsule size was measured as the difference between the total cell size and the cell body diameter and total cell size may be defined as the diameter of the complete cell including the capsule. The measurements were done with of at least 50 cells were measured using ImageJ 1.40 g software^1^ (National Institutes of Health, NIH, Bethesda, MD, United States) (Araujo Gde et al., 2012). Final measurements were presented as ratio of capsule size/cell diameter ratio. In addition, the surface-to-volume ratio (S/V) was calculated using the formula 3/*r*, where *r* is radius (Ferreira et al., 2015). Zeta potential (ZP) experiments were performed using Malvern Zetasizer Nano ZS equipment. The ZP was determined by Laser Doppler Micro-electrophoresis technique, at scattering angle of 173^o^, using a disposable cell folded capillary (DPS1060). ZP values were calculated as the average of ten independent measurements, each obtained as the mean of 30 counts (Nosanchuk et al., 1999).

### Isothermal Titration Calorimetry (ITC)

Isothermal titration calorimetry (ITC) experiments were carried out with one repetition using a VP-ITC microcalorimeter (Microcal LCC, Northampton, MA, United States) at 25°C, after previous electrical and chemical calibration. All the solutions employed in the experiment were previously degasified under vacuum (140 mbar) during 8 min. Each titration experiment consisted of 51 successive injections of 5 μL of eugenol at 1024 mg/L in a chamber containing 1.5 mL of *C. neoformans* suspension at 1 × 10^6^ CFU/mL. The first 1 mL injection was discarded to eliminate diffusion effects of material from the syringe to the calorimetric chamber. The injection time was 2 s and the interval between the injections was 240 s (Abraham et al., 2005; Raudino et al., 2011).

### Statistical Analyses

Results are shown as means ± SEM and *P-*values ≤ 0.05 were taken to indicate statistical significance. All statistical analyses were performed using GraphPad Prism version 5.00 for Windows (GraphPad Software, San Diego, CA, United States). Statistical significance among two groups was determined by Student’s *t-*test (parametric data) or Mann–Whitney test (non-parametric data) and multiple comparisons were analyzed by one-way analysis of variance (ANOVA). Zeta Plus software was used for Zeta potential (Brookhaven Instruments Corp., Holtsville, NY, United States).

## Results

### Antifungal Drug Susceptibility Testing, Time-Kill Curves, and Interaction between Eugenol and Antifungal Drugs

**FIGURE 1:**
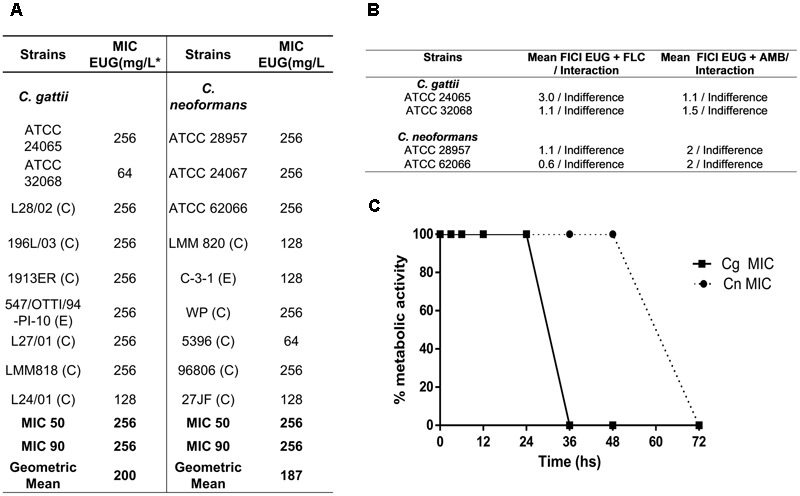
Table contents of minimum inhibitory concentration of eugenol (EUG) against the *Cryptococcus gattii* (Cg) and *C. neoformans* (Cn) strains using CLSI method **(A)**; values of interactions of antifungal drugs with eugenol Cg and Cn strains and **(B)**; and time-kill curves of EUG against *C. gattii* (filled squares) and *C. neoformans* (filled circles) **(C)**. Results of time-kill curve are expressed as the percentage of metabolic activity compared with the control growth. Data represent the mean of three independent experiments in duplicate assays. MIC, Minimal Inhibitory Concentration; C, Clinical; E, Environmental; EUG, Eugenol; FLC, Fluconazole, AMB, Amphotericin B; FICI, fractional inhibitory concentration index.

In the antifungal susceptibility testing, MIC range values for eugenol 64.0–256 (**Figure 1A**). To evaluate the kinetics of the action of the antifungal agents tested, the time-kill curve assay was performed. Eugenol provided fungicide curves, with complete reduction of growth after 36 h against *C. gattii* and 72 h against *C. neoformans*, both in MIC concentration (**Figure 1B**), and 2× MIC (data no shown). It was observed that there was no interaction between eugenol and the antifungal drugs fluconazole and amphotericin B against two *C. gattii* strains and two *C. neoformans* strains (**Figure 1C**).

### Sorbitol Test and Ergosterol Quantification

**FIGURE 2:**
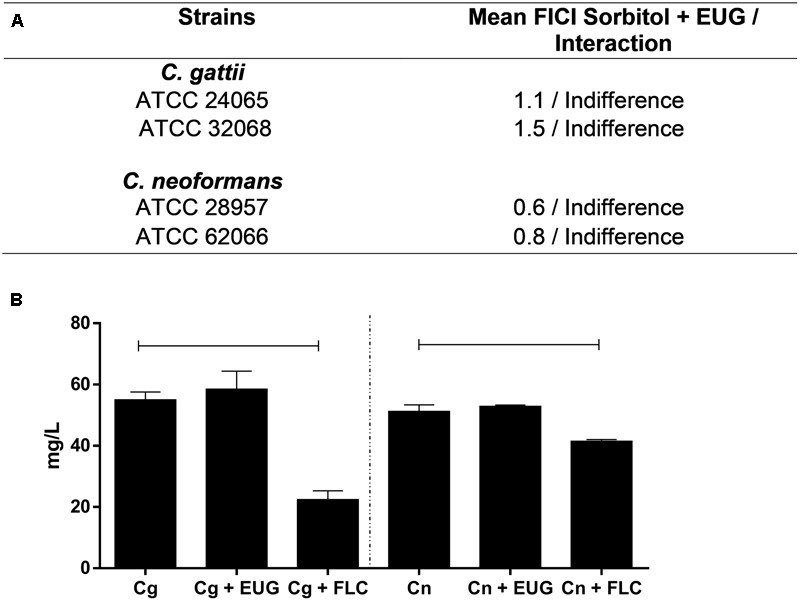
Checkboard of EUG and sorbitol to *C. gattii* and *C. neoformans* **(A)**; and levels of ergosterol content of Cg and Cn strains after 1 h of treatment with EUG and fluconazole (FLC; positive control). Results are expressed in mg/L. Statistically differences between the antifungal drugs and the control are represented by connect line (*P* < 0.05) **(B)**. Data represent the mean ± SEM of two independent experiments in triplicate assays. MIC, Minimal Inhibitory Concentration; EUG, Eugenol; FLC, Fluconazole, FICI, fractional inhibitory concentration index.

The results of the assays with sorbitol showed no alterations of MIC when the cryptococcal cells are exposed to the osmotic protector sorbitol (**Figure 2A**). It should be noted that after 1 h of treatment with eugenol treatment, in both *C. gattii and C. neoformans*, ergosterol levels were not reduced in comparison to control. The group that received fluconazole treatment (positive control) showed significantly reduced levels of ergosterol in the two cryptococcal species tested (*P <* 0.05) (**Figure 2B**).

### Mitochondrial Membrane Potential

**FIGURE 3:**
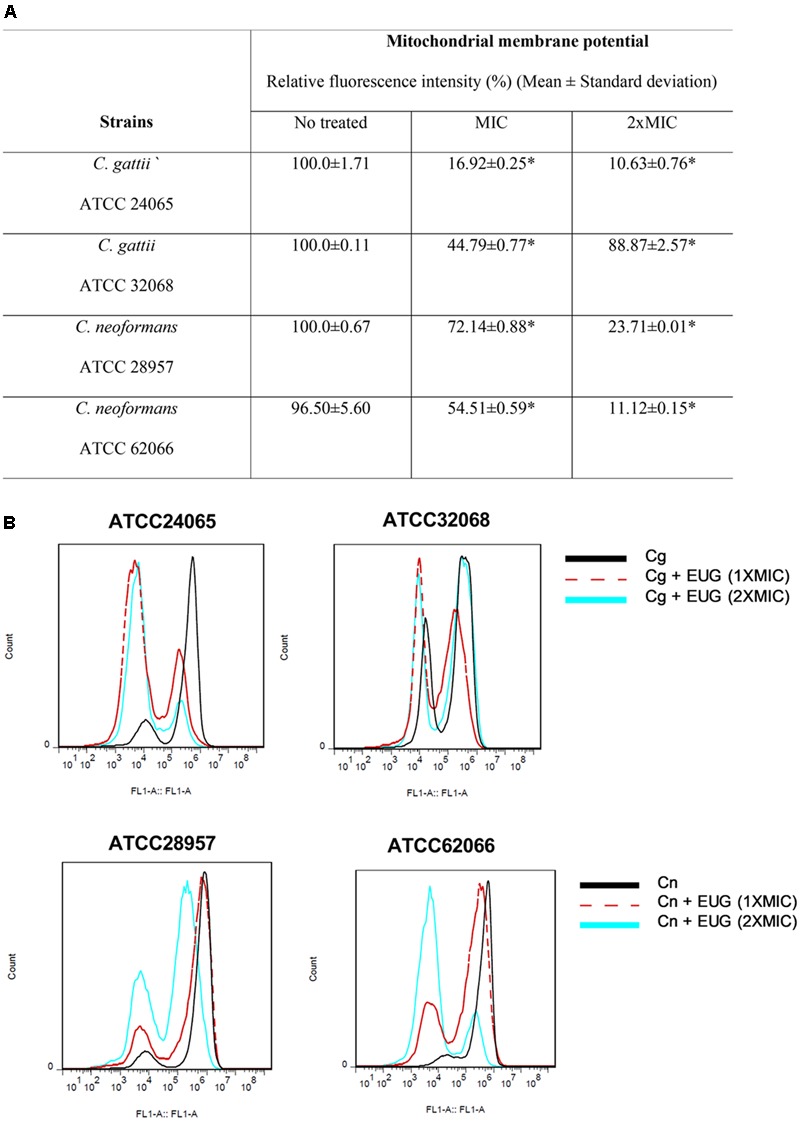
Eugenol induces mitochondrial membrane depolarization of *C. gattii* (Cg) and *C. neoformans* (Cn) cells after 12 h of treatment. Data are shown by table **(A)** and by histograms **(B)**. Statistically differences between the antifungal drugs and the control are represent by asterisks in table (*P* < 0.05). MIC, Minimal Inhibitory Concentration; EUG, Eugenol.

Next, we attempted to analyze the effects of eugenol in ΔΨm using an assay based on uptake and retention of Rho 123. The results from cells incubated for 1 h demonstrated no significant differences between eugenol-treated cells and cells with no treatment (**Supplementary Figure S1**). However, for 12 h of incubation, both concentrations of eugenol (MIC and 2× MIC) provoked mitochondrial depolarization as the Rho 123 fluorescence signal significantly decreased for all treated-strains when compared with control cells (*P* < 0.05) (**Figure 3**).

We observed relative fluorescence intensity (%) reduced to 16.92 ± 0.25 (MIC) and to 10.63 ± 0.76 (2× MIC) for *C. gattii* ATCC 24065. When *C. gattii* ATCC 32068 was analyzed, a smaller reduction was observed, with relative fluorescence intensities of 44.79 ± 0.77 and 88.27 ± 2.57 for the MIC and 2× MIC, respectively. For *C. neoformans* ATCC 28957, we observed a relative fluorescence intensity (%) reduced to 72.14 ± 0.88 (MIC) and to 23.71 ± 0.01 (2× MIC). And for *C. neoformans* ATCC 62066, a bigger reduction was observed, with relative fluorescence intensities of 54.51 ± 0.59 and 11.12 ± 0.15 for the MIC and 2× MIC, respectively (**Figure 3**).

### Lysosomal Membrane Stability

**FIGURE 4:**
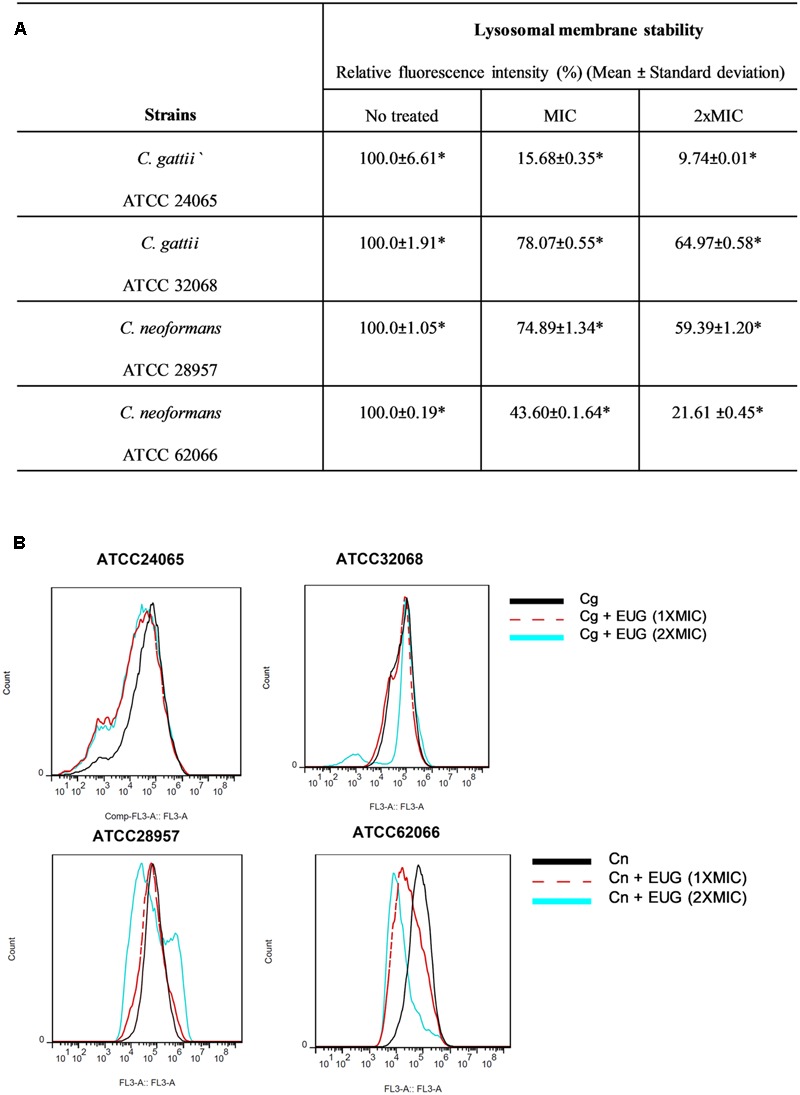
Eugenol reduces the lysosomal integrity of *C. gattii* (Cg) and *C. neoformans* (Cn) cells after 12 h of treatment. Data are shown by table **(A)** and by histograms **(B)**. Statistically differences between the antifungal drugs and the control are represent by asterisks in table (*P* < 0.05). MIC, Minimal Inhibitory Concentration; EUG, Eugenol.

The functionality of yeast lysosomes after eugenol treatment was evaluated using AO staining followed by flow cytometry analysis and microscopy fluorescence. Untreated cells exhibited intact lysosomes marked by a strong fluorescence emission in the FL3 channel. The results from cells incubated for 1 h demonstrated no significant differences between eugenol-treated cells and cells with no treatment (**Supplementary Figure S2**). Eugenol significantly reduced the lysosomal integrity (*P <* 0.05) (**Figure 4**).

We observed relative fluorescence intensity (%) reduced to 15.68 ± 0.35 (MIC) and to 9.74 ± 0.01 (2xMIC) for *C. gattii* ATCC 24065. When *C. gattii* ATCC 32068 was analyzed, a smaller reduction was observed, with relative fluorescence intensities of 78.07 ± 0.55 and 64.97 ± 0.58 for the MIC and 2× MIC, respectively. For *C. neoformans* ATCC 28957, we observed a relative fluorescence intensity (%) reduced to 74.89 ± 1.34 (MIC) and to 59.39 ± 1.20 (2× MIC). And for *C. neoformans* ATCC 62066, a bigger reduction was observed, with relative fluorescence intensities of 43.60 ± 1.64 and 21.61 ± 0.45 for the MIC and 2× MIC, respectively (**Figure 4**).

### Lipid Peroxidation and ROS Production

**FIGURE 5:**
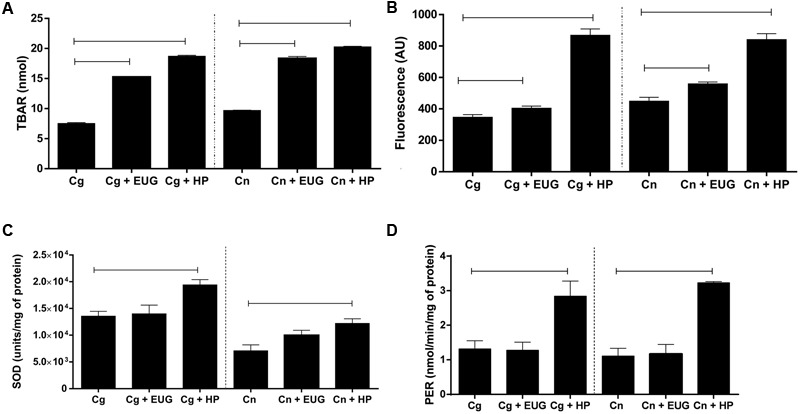
Lipid peroxidation **(A)**, amounts of reactive oxygen species (ROS) **(B)**, superoxide dismutase (SOD) **(C)**, and peroxidase (PER) **(D)** activities in *C. gattii* and *C. neoformans* strains after 1 h of treatment with eugenol (EUG) and hydrogen peroxide (HP; positive control). Lipid peroxidation results are expressed in nmol, ROS in arbitrary units (AU) of fluorescence, SOD in units/mg of protein and PER in nmol/min/mg of protein. Statistically differences the treatments are represent by connect line (*P* < 0.05). Data represent the mean ± SEM of two independent experiments in triplicate assays. MIC, Minimal Inhibitory Concentration; EUG, Eugenol; HP, Hydrogen Peroxide.

Our results showed high levels of TBARS after 1 h treatment with eugenol (*C. gattii* – no treated: 7.52 ± 0.40 nmol, treated: 15.24 ± 0.15 nmol; *C. neoformans* – no treated: 9.68 ± 0.13 nmol, treated: 18.33 ± 0.86 nmol) with the growth control (*P* < 0.05) and hydrogen peroxide (positive control), indicating damage of the lipids compared (**Figure 5A**). It is well established that lipid peroxidation is mediated by free radicals. Thus, it was hypothesized that this effect may be related to ROS. Eugenol resulted in a significantly increasing ROS levels compared with the growth control after 1 h of treatment (*C. gattii* – no treated: 347.00 ± 33.31 AU, treated: 404.06 ± 26.24 AU; *C. neoformans* – no treated: 449.60 ± 59.99 AU, treated: 560.00 ± 27.98 AU) (*P* < 0.05) (**Figure 5B**).

### Antioxidant PER and SOD Activities

To determine whether treatment with eugenol influences the antioxidant enzymes, activities of SOD and PER were assessed. The results showed that is no difference in SOD (*C. gattii* – no treated: 13568.00 ± 899.80 units/mg of protein, treated: 14016.00 ± 4581.00 units/mg of protein; *C. neoformans* – no treated: 7099.00 ± 1097.00 units/mg of protein, treated: 9970.00 ± 947.20 units/mg of protein) (*P* < 0.05) (**Figure 5C**) and PER (*C. gattii* – no treated: 1.32 ± 0.23 nmol/min/mg of protein, treated: 1.28 ± 0.23 nmol/min/mg of protein; *C. neoformans* – no treated: 1.11 ± 0.38 nmol/min/mg of protein, treated: 1.17 ± 0.27 nmol/min/mg of protein) (*P* < 0.05) (**Figure 5D**) activities in cells exposed to eugenol when compared to the growth control.

### Morphological Alterations

**FIGURE 6:**
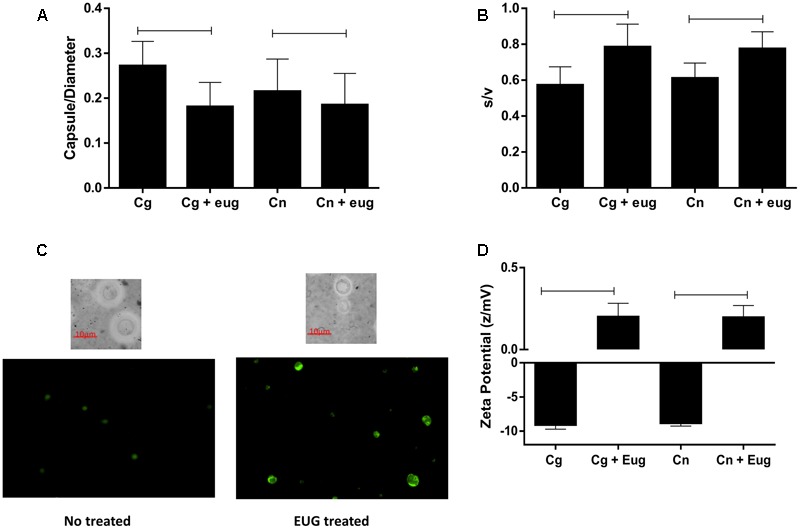
Morphometric parameters and cellular charge are altered by the eugenol stress in *C. gattii* and *C. neoformans* strain. Capsule size/diameter **(A)**; surface/volume **(B)**; stained cells (India ink and acridine orange, AO) **(C)**, and zeta potential **(D)** of cryptococcal cells treated with eugenol and with no stress. Morphometric data are expressed in ratio and zeta potential data are expressed in z/mV. Statistically differences the treatments are represent by connect line (*P* < 0.05). Data represent the mean ± SEM of two independent experiments in triplicate assays. MIC, Minimal Inhibitory Concentration; EUG, Eugenol; FLC, Fluconazole; FICI, fractional inhibitory concentration index.

Morphometric analysis showed that eugenol reduces significantly capsule size/cell diameter ratios (*C. gattii* – no treated: 0.27 ± 0.005, treated: 0.18 ± 0.005; *C. neoformans* – no treated: 0.21 ± 0.007, treated: 0.18 ± 0.006) (*P* < 0.05) (**Figure 6A**) and surface/volume (s/v) ratios (*C. gattii* – no treated: 0.57 ± 0.01, treated: 0.78 ± 0.01; *C. neoformans* – no treated: 0.61 ± 0.008, treated: 0.78 ± 0.009) (*P* < 0.05) (**Figure 6B**) of *C. gatii* and *C. neoformans*. Examples of images that support the data are in the **Figure 6C**. Moreover, using a zeta potential analyzer it could be noted that eugenol treatment caused an increase of the cellular superficial charges in both *C. gattii* and *C. neoformans* (*C. gattii* – no treated: -9.17 ± 01.32 z/mV, treated: 0.21 ± 0.19 z/mV; *C. neoformans* – no treated: -8.91 ± 0.35, treated: 0.20 ± 0.17) (*P* < 0.05) (**Figure 6D**).

As a means of studying lysosomal membrane integrity in yeasts, we recorded imagens in a fluorescent microscopy from stained cells with AO using 495–555 nm filter. Treatment of eugenol promote a loss of the lysosomal pH gradient and subsequent leakage of AO into the cytosol (Petersen et al., 2010), that results in rise in green fluorescence when we compared with cells no treated with eugenol (**Figure 6C**).

### Thermodynamics of the Interaction of Eugenol with *C. neoformans*

**FIGURE 7:**
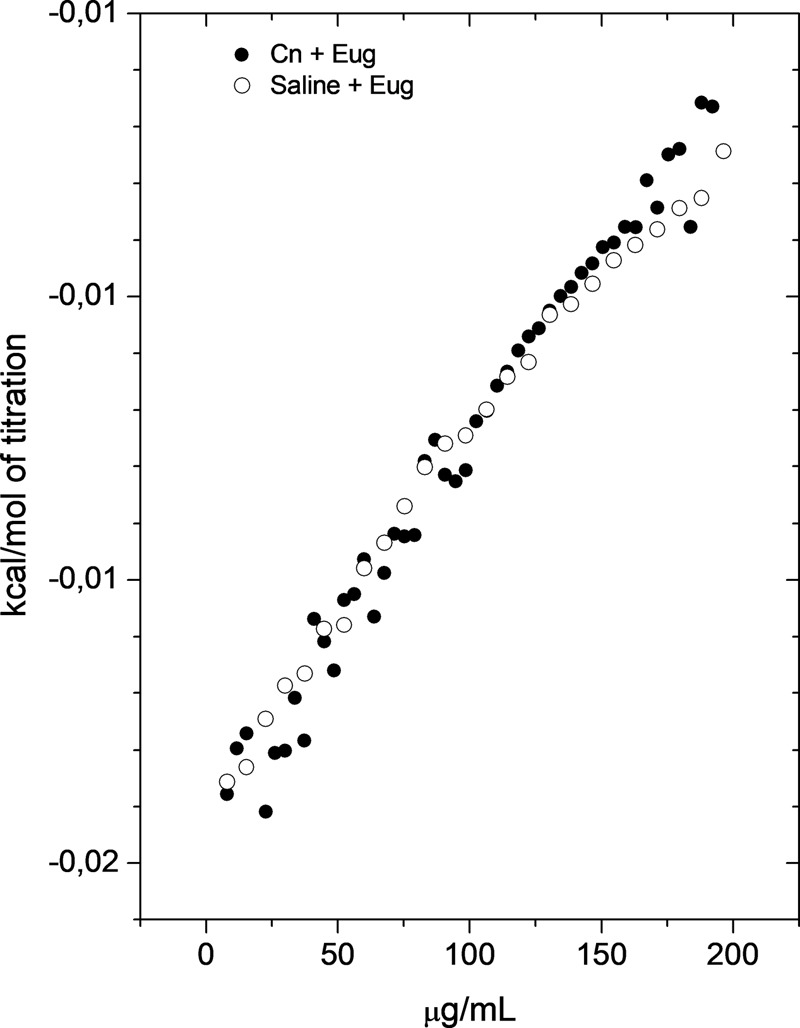
*Cryptococcus neoformans* did not interact significantly with eugenol. Calorimetric titration curve for the dilution of concentrated EUG into saline solution (control) (○) and *C. neoformans* cells (●). Each titration experiment consisted of 51 successive injections of 5 μL of eugenol at 1024 mg/L in 1.5 mL of *C. neoformans* suspension at 1 × 10^6^ CFU/mL.

Aiming to analyze the molecular interactions between eugenol and cryptococcal cells, enthalpy changes of this process were determined by ITC. **Figure 7** shows that *C. neoformans* did not interact significantly with eugenol, since the enthalpy values of the group that received eugenol treatment were similarly of the blank experiment (eugenol in saline).

## Discussion

A small number of antimycotic drugs with different modes of action and target spectra are available to treat cryptococcosis, with polyenes and azoles being the most used (Chayakulkeeree and Perfect, 2006). It is well established that the infection caused by *C. gattii* has a less favorable response to antifungal therapy and a relatively worse prognosis compared with the infection caused by *C. neoformans* (Kronstad et al., 2011). The rapid increase in the emergence of resistance against existing antifungal drugs created a need to discover new antifungal agents. Previous studies showed that eugenol has antifungal activity against *C. neoformans*, however, little is known about its activity against *C. gattii* and about its mechanism of action (Lemos et al., 2005; Carrasco et al., 2012). In this study, we provide evidence that eugenol has antimicrobial activity against *C. gattii*. Also, the present study gives a clear understanding and further validates a possible mode of action of eugenol against *C. gattii* and *C. neoformans*.

The results of the *in vitro* susceptibility tests of the fungal isolates to eugenol, main constituent of essential oil of *Ocimum gratissimum*, presented MIC values range from 64 to 256 mg/L for both species. Similar results were found in previous studies for *C. neoformans* (Lemos et al., 2005; Carrasco et al., 2012). It is important to note that after combining fluconazole and amphotericin B, eugenol exert indifferent effect against *C. gattii* and *C. neoformans in vitro*. These results suggest that eugenol could be used together with fluconazole and amphotericin B for cryptococcosis treatment without prejudice to the action of these drugs, but more studies are necessary to confirm this hypothesis.

To provide clues if eugenol interferes on fungal cell-wall integrity, a checkerboard microdilution method was conducted with eugenol and sorbitol, an osmotic protectant used for stabilizing fungal protoplasts (Lemos et al., 2005; Carrasco et al., 2012). Results showed that MIC of eugenol did not vary in the presence of sorbitol, but more tests are necessary to prove this hypothesis.

Our results showed that eugenol does not disturb the amount of ergosterol in cryptococcal cells. Khan et al. (2013) found contradictory results using similar methodology for *C. albicans* when they studied the influence of eugenol on ergosterol and on cell wall. Otherwise, Carrasco et al. (2012) showed that the antifungal activity of eugenol derivative against *C. albicans* was not reversed in the presence of an osmotic support such as sorbitol, and does not seem to bind to ergosterol. We think that the data found for cryptococcal cells could be attributed to its encapsulated form. The capsular polysaccharide confers several physicochemical properties to the cell surface, including a negative surface charge and a hydrophilic surface (Araujo Gde et al., 2012), that could disturb eugenol mechanism of action.

High levels of TBARSs were observed after 1 h treatment with eugenol. Our results also show that eugenol caused an increase in intracellular amount of ROS. Based on these data, it was hypothesized that eugenol induces ROS production. Under dysfunctional mitochondria, electrons can escape from the electron transport chain to induce formation of superoxide anions by one-electron reduction of oxygen. In this, study it was shown that the presence of eugenol can contribute substantially to the generation of ROS, possibly triggered by disturbance in the mitochondrial membrane and a reduction of a lysosomal integrity. This event seems to be connected to the potential of eugenol to interfere with electronic mitochondrial membrane stabilization. Furthermore, the phenomenon of depolarization of the mitochondrial membrane can be evidenced by increased generation ROS, as seen by vanillin derivatives against *C. neoformans* (Kim et al., 2014). In addition, leading to lipid peroxidation, which corresponds to the oxidative degradation of lipids, in which a free radical chain ‘steals’ electrons from the lipids (mainly polyunsaturated fatty acids) in cell membranes, resulting in cell damage. Similar results were found for eugenol against *C. albicans* (Khan et al., 2013).

To better understand how the cryptococcal cells adapt to eugenol stress, we performed some experiments exposing the yeasts in sub inhibitory concentration of eugenol (0.5× MIC). The assays demonstrated no difference on SOD and PER activities. The antioxidant system cell was not sufficient to neutralize the ROS induced by eugenol. It could explain the fungicidal action seen in time-kill curve.

Eugenol stress altered the cell morphology and induced a reduction in capsule size/cell diameter ratio and an increase in the surface/volume ratio. Similar results have been reported following the exposure of *C. neoformans* to fluconazole, voriconazole, amphotericin B, and terbinafine (Mondon et al., 1999; Nosanchuk et al., 1999; Guerra et al., 2012; Santos et al., 2014). Our group previously demonstrated that itraconazole promotes oxidative burst, and the cells adapt to this process diminishing the capsule size and cell diameter (Ferreira et al., 2013, 2015). This phenomenon could be explained as an attempt of the cells to adapt to different xenobiotics, since the capsule growth is associated to a slower yeast growth. Indeed, cells with high surface/volume ratio cells adapt more rapidly to abrupt changes in environmental conditions (Maxson et al., 2007; Ferreira et al., 2015).

Cryptococcal cells are negatively charged because of capsular polysaccharide and melanin (Kozel, 1983). As expected, the cells growth in the presence of eugenol decreased the magnitude of the negative charge, because they have smaller capsule than yeasts exposed to no stress. Based on this observation, we hypothesized that cells treated with eugenol would be more phagocytized by macrophages due to reduced electrostatic repulsion between yeast cells and phagocytes, but more studies are necessary to confirm this hypothesis.

To better understand the chemical nature of interaction between eugenol and superficial yeast molecules, we performed ITC. The analysis of this molecular interaction showed interaction with slow significant force. Hydrogen bonds, ionic interactions, covalent interactions are interactions that have a power of about 1.0 to 10 kcal/mol. The non-polar interactions like and hydrophobic and induced dipole–dipole (Van der Walls) have energy lower than 1 kcal/mol and generate low enthalpy connection (Berg et al., 2002). Based on these informations, we supposed that eugenol interacts with cryptococcal cells through non-polar interactions.

## Conclusion

The present results indicate that eugenol has antifugal effects against *C. gattii* and *C. neoformans* acting on the oxidative burst. It is known that eugenol is able to pass the blood–brain barrier, enter the brain and act *in situ* (Bahramsoltani et al., 2015). In this way, eugenol could be a possible molecule to the treatment of cryptococcosis, and could have its activity improved through organic modeling. Indeed, eugenol could be used as an auxiliary treatment to cutaneous cryptococcosis or another cutaneous mycosis (Chami et al., 2004; Lee et al., 2007).

## Author Contributions

JA, ÁS, BM, LVNFS: Execution, analysis, and interpretation of data for the tests about morphologic cell surface, ergosterol content, cellular wall integrity, lipid peroxidation, oxidative burst from the interaction of eugenol with cryptococcal cells. JS, LCNS, JR, WN, EF: Execution, analysis, and interpretation of data for the tests about lysosomal and mitochondrial integrity from the interaction of eugenol with cryptococcal cells. GF, DS, AM, VdS, AP, TR, LG: Conception or design of all the work, preparation of the article, and translation into English. ÂD: Execution, analysis, and interpretation of data for the tests about the thermodynamics changes from the interaction of eugenol with cryptococcal cells.

## Conflict of Interest Statement

The authors declare that the research was conducted in the absence of any commercial or financial relationships that could be construed as a potential conflict of interest.

## Footnotes

1 http://rsb.info.nih.gov/ij/

## Supplementary Material

FIGURE S1 Mitochondrial membrane depolarization of *C. gattii* (Cg) and *C. neoformans* (Cn) cells. Cg ATCC 24065 strain **(A,B)**; Cg ATCC 32068 strain **(C,D)**; Cn ATCC 28957 strain **(E,F)**; Cn ATCC 62066 **(G,H)** strain after 1 h of treatment with eugenol. Data are shown by column graphs (mean ± SE) and by histograms. MIC, Minimal Inhibitory Concentration; EUG, Eugenol.

FIGURE S2 Lysosomal integrity of *C. gattii* (Cg) and *C. neoformans* (Cn) cells. Cg ATCC 24065 strain **(A,B)**; Cg ATCC 32068 strain **(C,D)**; Cn ATCC 28957 strain **(E,F)**; Cn ATCC 62066 **(G,H)** strain after 1 h of treatment with eugenol. Data are shown by column graphs (mean ± SE) and by histograms. MIC, Minimal Inhibitory Concentration; EUG, Eugenol.
