# Supplementary figures and images for: Eugenol Induces Phenotypic Alterations and Increases the Oxidative Burst in Cryptococcus
Source: Front Microbiol. 2017 Dec 7;8:2419. doi: 10.3389/fmicb.2017.02419 (PMC5726113; doi:10.3389/fmicb.2017.02419)

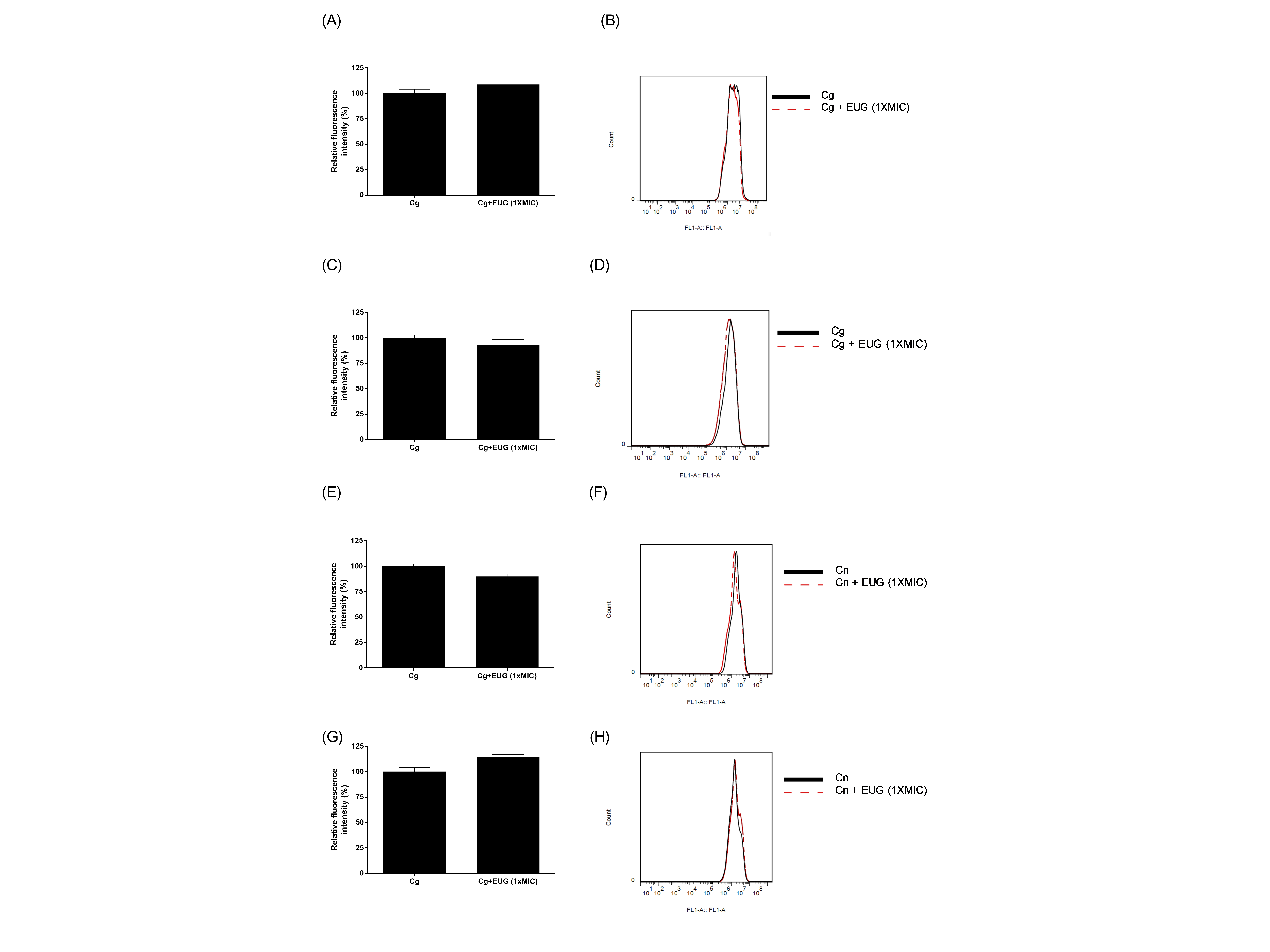

Supplement: FIGURE S1 — Mitochondrial membrane depolarization of C. gattii (Cg) and C. neoformans (Cn) cells. Cg ATCC 24065 strain (A,B); Cg ATCC 32068 strain (C,D); Cn ATCC 28957 strain (E,F); Cn ATCC 62066 (G,H) strain after 1 h of treatment with eugenol. Data are shown by column graphs (mean ± SE) and by histograms. MIC, Minimal Inhibitory Concentration; EUG, Eugenol. [file Image_1.TIF]

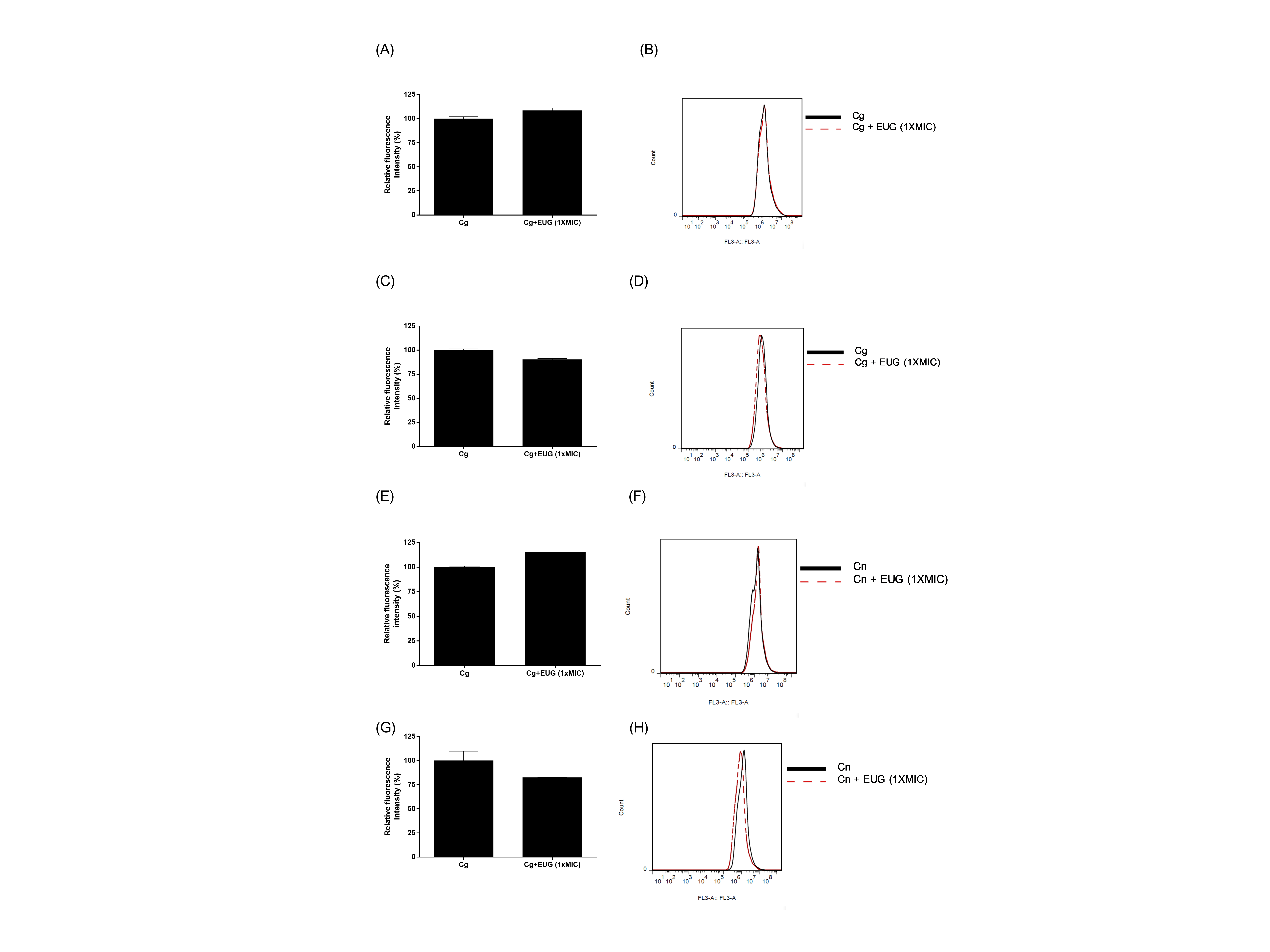

Supplement: FIGURE S2 — Lysosomal integrity of C. gattii (Cg) and C. neoformans (Cn) cells. Cg ATCC 24065 strain (A,B); Cg ATCC 32068 strain (C,D); Cn ATCC 28957 strain (E,F); Cn ATCC 62066 (G,H) strain after 1 h of treatment with eugenol. Data are shown by column graphs (mean ± SE) and by histograms. MIC, Minimal Inhibitory Concentration; EUG, Eugenol. [file Image_2.TIF]
